# A Multi-Streamline Approach for Upcycling PET into a Biodiesel and Asphalt Modifier

**DOI:** 10.3390/polym16060796

**Published:** 2024-03-13

**Authors:** Kainan Chen, Zeinab Mraiza, Yunqiao Pu, Jinghao Li, Zhihua Liu, Arthur J. Ragauskas, Fujie Zhou, Joshua S. Yuan

**Affiliations:** 1 Synthetic and Systems Biology Innovation Hub, Department of Plant Pathology and Microbiology, Texas A&M University, College Station, TX 77843, USA; ckainan@wustl.edu (K.C.); jinghaol@wustl.edu (J.L.); zhliu@tamu.edu (Z.L.); 2 Texas A&M Transportation Institute, Texas A&M University, College Station, TX 77843, USA; zainabrabeea@gmail.com; 3 Joint Institute for Biological Sciences, Biosciences Division, Oak Ridge National Laboratory, Oak Ridge, TN 37831, USA; puy1@ornl.gov (Y.P.); aragausk@utk.edu (A.J.R.); 4 Department of Chemical and Biomolecular Engineering, The University of Tennessee, Knoxville, TN 37996, USA; 5 Department of Forestry, Wildlife and Fisheries, Center for Renewable Carbon, Institute of Agriculture, The University of Tennessee, Knoxville, TN 37996, USA; 6 Department of Energy, Environmental, and Chemical Engineering, McKelvey School of Engineering, Washington University in St. Louis, St. Louis, MO 63130, USA

**Keywords:** PET upcycling, plastic waste valorization, asphalt binder, infrastructure resilience

## Abstract

The non-degradable nature of petroleum-based plastics and the dependence on petroleum-based products in daily life and production are dilemmas of human development today. We hereby developed a plastic waste upcycling process to address these challenges. A multi-stream fraction strategy was developed to process poly (ethylene terephthalate) (PET) plastics into soluble and insoluble fractions. The soluble fraction was used as a sole carbon source for microbial fermentation to produce biodiesel precursor lipids with an appreciable bioconversion yield. The insoluble fraction containing fractionated polymers was used as the asphalt binder modifiers. The downsized PET additive improved the high-temperature performance of the asphalt binder by 1 performance grade (PG) without decreasing the low-temperature PG. Subsequent SEM imaging unveiled alterations in the micromorphology induced by PET incorporation. Further FTIR and ^1^H NMR analysis highlighted the aromatic groups of PET polymers as a crucial factor influencing performance enhancement. The results demonstrated the multi-stream fraction as a promising approach for repurposing plastic waste to produce biodiesel and modify asphalt. This approach holds the potential to tackle challenges in fuel supply and enhance infrastructure resilience to global warming.

## 1. Introduction

Contemporary society is facing two prominent issues: the accumulation of plastic waste and the emission of global warming [1,2]. There has been an unprecedentedly rapid rise in plastic waste due to the massive demand for plastic products in various industrial and consumer products [3]. Poly (ethylene terephthalate) (PET) is one of the largest groups of plastics, with about 70 million tons manufactured and used each year [4]. Due to their resistance to natural degradation, PET plastics can remain in the ecosystem for decades, causing environmental pollution and impacting public and environmental health [5]. Furthermore, insufficient and unsustainable practices in the management and treatment of plastic can also contribute to global warming [6,7].

The inexorable progression of global warming and climate change draws us closer to increasingly uncertain and potentially catastrophic climate impacts, which will have substantial consequences for transportation infrastructure [8]. In particular, the rise in temperature resulting from global warming contributes to elevated the thermal stresses and escalated corrosion of steel structures in infrastructure, as well as the reduced stiffness of flexible pavements, disrupting the proper distribution of loads [9,10,11]. Therefore, infrastructure with improved resistance to higher temperature will be crucial to ensure the efficiency of transportation networks in the context of future global warming [12]. Among the transportation system, asphalt pavement is highly sensitive to the temperature conditions [13,14]. Studies have shown that high-temperature-induced permanent deformation is a primary concern for asphalt pavements in climate warming scenarios [15,16]. Improving the high-temperature performance of asphalt materials constitutes a primary measure for addressing deformation caused by temperature. The modification of asphalt or the incorporation of additives can augment the resistance of asphalt mixtures to permanent deformation to a certain degree [17,18,19].

In recent years, plastic waste has been increasingly studied as an additive to asphalt in recent years [3]. Many works have reported that the addition of plastic improves the mechanical properties of asphalt [20,21,22,23,24,25,26,27,28,29]. Moreover, there are emerging studies showing that PET particle-modified asphalt shows improved high-temperature performance [5,30,31]. Compared to coarse PET particles, fine PET or PET small particle sizes show improved modification effects since the smaller sizes can create a more uniform distribution of PET particles in asphalt [5,30,31]. Finely ground PET is commonly produced through grinding or crushing processes, leading to the generation of microparticles. This practice poses a potential environmental concern due to the negative impact associated with the interactions of microplastics throughout the ecosystem [32,33]. A treatment method to downsize the PET particles in a way that is both efficient and environment-friendly is thus important for PET waste upcycling and its applications in asphalt modification.

**Figure 1:**
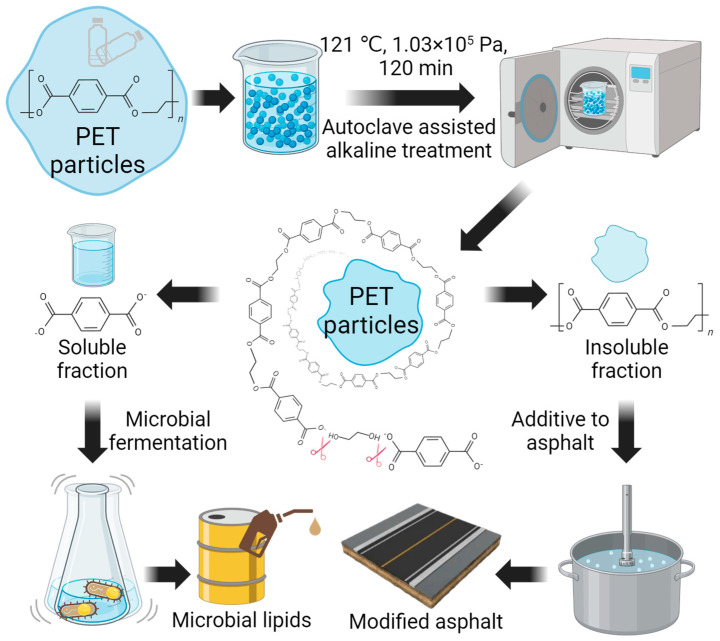
Schematics of alkaline-assisted fractionation and multi-stream usage of plastic PET. The picture is created with BioRender.com.

Our previous research has established a hydrolysis-based fractionation process for lignin and found that fractionated lignin can improve the high-temperature property of asphalt binder modifier without compromising the low-temperature property [34]. Considering that both PET and lignin are aromatic polymers, we explored a PET fractionation process to downsize PET particles for asphalt binder additive usage. Unlike other solvolysis-based methods like methanolysis and aminolysis, the hydrolysis process allows for the depolymerization of PET in water without the introduction of extra substances such as methanol and amines, which enhances its environmental and economic viability [35]. In this study, we developed a fractionation strategy based on alkaline hydrolysis to reduce the molecular weight of PET and turn it into soluble and insoluble parts for different applications (Figure 1). We further demonstrated that the insoluble fraction improved the high-temperature performance of the asphalt binder modifier without significantly compromising its low-temperature performance, and the soluble fraction is amenable to microbial fermentation and the production of biodiesel precursor lipids.

## 2. Materials and Methods

### 2.1. Matreials

The PET powder (catalog number ES306031) was purchased from Goodfellow Corporation (Coraopolis, PA, USA). All chemicals were purchased from Sigma-Aldrich, (MilliporeSigma, Burlington, MA, USA).

### 2.2. Methods

#### 2.2.1. PET Fractionation via Alkali-Heat Treatment

A suspension comprising 10% (*w/v*) PET powders and 1% (*w/v*) NaOH was prepared in deionized water. The mixture underwent autoclaving for 2 h at a temperature of 121 °C and a pressure of around 15 pounds per square inch (103,421 Pa). The solubilized PET and insolubilized fraction were separated by centrifuging at 8000× *g* for 10 min and collected. Following the fractionation process, the insoluble fraction of PET was subjected to two washes with deionized water. Subsequently, it was dried to a constant weight in an oven set at 80 °C for future utilization as an additive to asphalt binders. The solubilized PET fraction underwent precipitation through the addition of HCl to eliminate the alkali solution. The resulting white precipitate was collected by centrifugation at 8000× *g* for 10 min, followed by two washes with deionized water. Subsequently, the washed precipitate (the soluble fraction) was dried to a constant weight in an oven set at 80 °C for future utilization as a carbon source in microbial fermentation.

#### 2.2.2. Characterization of PET Insoluble Fraction

The morphology and size of the PET particles were investigated utilizing an optical microscope (Boreal Science, St. Catharines, ON, Canada). Under bright-field conditions, visible light illuminated the sample, and images of particles with a scale were captured by a digital camera.

#### 2.2.3. Characterization of PET Soluble Fraction

The previously reported sample preparation method was adopted with modifications [36]. Specifically, a 2 mL solubilized PET fraction was transferred into screw-cap reaction vials. Acid hydrolysis was performed by adding 300 μL of HCl (to pH 1–2). Samples were extracted twice with 2 mL of ethyl acetate by agitating for 15 min and centrifuging for 10 min at 2500 rpm. Upper organic layers were transferred into new glass tubes. Extracts were evaporated to dryness under a gentle nitrogen stream at 40 °C. Residues were resuspended in 300 μL of ethyl acetate; 30 μL of BSTFA were added for derivatization. Specifically, the samples were heated at 60 °C for 60 min to convert total terephthalic acid (TPA) equivalents into 1,4-bis(trimethylsilyl)terephthalic acid by replacement of the hydrogen of the hydroxy groups. Derivatized extracts were then cooled to room temperature for gas chromatography/mass spectrometry (GC/MS) analysis.

GC/MS-QP2010SE, (Shimadzu Scioentifc Instruments, Inc., Houston, TX, USA) equipped with a Zebron ZB-5HT Inferno column (30 m × 250 µm ID × 0.1 µm df) was used for analysis. The injector was set to a temperature of 260 °C, the transfer line was at 250 °C, the ion source was at 230 °C, and a constant column flow rate was held at 1.0 mL of helium/min. The oven temperature program started at 200 °C for 3 min, increased to 260 °C at 30 °C/min, was held for 11 min, then increased to 280 °C at 35 °C/min, and was held for 4 min. The mass detector operated in single ion monitoring mode, using a 70 eV electron beam to generate fragment ions. The spectrum was analyzed using the workstation software (version 4.11 SU2) in the GC/MS system to identify the derivatives of the PET soluble fraction.

#### 2.2.4. Microbial Fermentation Using Fractionated Soluble PET

The soluble fraction of PET was used as the sole carbon source for microbial fermentation. Briefly, the seed culture of *Rhodococcus jostii* RHA1 was grown at 30 °C in LB broth to an OD_600_ of about 0.8. One percent of the seed culture was inoculated into the minimal salt medium [37] containing 3.2 g/L of dried fractionated PET (from the soluble part) as the sole carbon source. Cultures were incubated at 30 °C with shaking at 200 rpm for 5 days. Biomass increase was monitored by measuring OD_600_. At the end of fermentation, all 50 mL of the cultures were collected by centrifuging at 5000× *g* for 10 min to collect RHA1 cells for lipid characterization.

#### 2.2.5. Lipid Extraction, Identification, and Quantification

Briefly, the RHA1 cell pellets were harvested by centrifugation at 5000 rpm for 10 min and subsequently lyophilized for 48 h. The total dry cell weight (DCW) of each sample was measured with analytic balance (Sartorius, Ann Arbor, MI, USA). An amount of 5–10 mg of the dried biomass pellets was incubated with 2 mL of methanol–sulfuric acid (*v/v* = 85:15) solution and 2 mL of chloroform at 95 °C for 180 min, during which the acid catalyzed the methanolysis, converting the fatty acids of the lipids into their corresponding monomeric derivatives that were dissolved in the chloroform. After incubation, the sample was moved from the oven and cooled to room temperature. Then, 1 mL of deionized water was added twice for washing until no acid residual remained. The upper layer (water phase) was removed using a vacuum system to collect the organic layer. Two hundred microliters of the organic section were diluted in 1800 µL of chloroform, which contained internal standard methyl benzoate at a concentration at 21 µg/mL. The well mixed solution was then filtered using a 0.2 µm filter before GC/MS analysis.

GC/MS (QP2010SE, Shimadzu) equipped with a Zebron ZB-35HT Inferno column (30 m × 250 µm ID × 0.25 µm df) was used for analysis. Helium was used as a carrier gas at a 1.0 mL/min linear velocity. The injector was set to a temperature of 260 °C, the transfer line was at 250 °C, and the ion source was at 230 °C. The gas chromatograph (GC) oven temperature program was set initially at 50 °C for 3 min and then increased to 300 °C at 10 °C/min. The mass detector operated using a 70 eV electron beam with an ionization current of 40 μA. The monomeric derivatives of lipids were identified using the Compound Composer Database Software in the GC/MS system.

The quantification for each component was performed by calculating a response factor (RF) for each component analytical standard. For example, the RF for C16 monomer analytical standard was calculated using the following expression:

(1) RF=(Ax×Cin)/(Ain×Cx)=(Ax/Ain)/(Cx/Cin)

where Ax is the peak area of the C16 analytical standard, Ain is the peak area of the methyl benzoate internal standard, and Cx and Cin are the concentrations of the C16 analytical standard and methyl benzoate internal standard, respectively. Based on the peak area and concentration of the internal standard, as well as the peak area of C16, the concentrations of C16 monomer in the 2 mL GC/MS sample were determined. The weight of lyophilized cells used for lipid extraction was then employed to calculate the lipid content in the RHA1 cells.

#### 2.2.6. Characterizing Asphalt Binder Temperature Performance Grade

Fractionation PET (insoluble part) were incorporated into the pre-melt asphalt binder at concentrations of 2% and 5% (*w/w*). The mixture was thoroughly stirred. High-temperature performance grade characterization was conducted using AASHTO T315 [38]. Briefly, the Dynamic Shear Rheometer (DSR) test was carried out on 25 mm diameter specimens, and the temperature at which rutting parameters (G*/sin δ) of the asphalt binder specimens equal to 1.0 kPa was determined, a value typically indicating the onset of softening or reduced resistance of asphalt binder specimens to rutting or permanent deformation [39]. The measured temperature is defined as high critical temperature [40]. AASHTO T313 was used to characterize the low temperature performance grade [41].

Briefly, asphalt binders were aged with a two-step process, including a rolling thin-film oven (RTFO) at 163 °C for 85 min and a pressure aging vessel (PAV) at 100 °C, 2.1 MPa, for 20 h [42]. The long-term aged asphalt binders were heated and molded to beam specimens. The Bending Beam Rheometer (BBR) test was carried out to determine the temperature in a condition of the parameters of relaxation constant (m) and flexural creep stiffness (S) at 60 s of loading equal to 0.300 and 300 kPa, respectively. Typically, these two values are critical points in the BBR test where the asphalt binder begins to lose some of its viscoelastic properties and may exhibit deformation under the applied load [43]. The determined temperature was defined as low critical temperature and served as the indicator for low temperature performance. The high and low temperature performance grades of all tested asphalt binders were accordingly determined by the results for DSR and BBR tests according to the AASHTO M320 specification [44].

#### 2.2.7. Scanning Electron Microscope (SEM) Characterization

Fractionated PET-modified asphalt binders were prepared by melting at 150 °C and mounted on samples stages with carbon tape and coated with a thin layer of gold to enhance conductivity. Samples were observed using a TESCAN LYRA-3 Model GMH Focused Ion Beam Microscope (KEYENCE Corp. of America, Itasca, IL, USA). The images were collected at a working distance of 10 mm. The accelerating voltage applied was 5 kV.

#### 2.2.8. Fourier-Transform Infrared Spectroscopy (FTIR) Characterization

Fractionated PET-modified asphalt binders were analyzed using a Nicolet i50 FTIR spectrometer (Thermo Fisher Scientific, Houston, TX, USA). FTIR spectra of all samples were collected using an attenuated total reflection (ATR) stage. Samples were loaded in ATR crystal. All samples were scanned 64 times and acquired at a spectral resolution of 4 cm^−1^.

#### 2.2.9. Proton Nuclear Magnetic Resonance (^1^H NMR) Characterization

The ^1^H NMR spectra of original and treated samples were recorded on a Bruker Avance III 400-MHz spectrometer (Bruker Scientific LLC, Billerica, MA, USA) with a broadband inverse (BBI) probe. The asphalt samples were dissolved in 0.75 mL of deuterated chloroform (CDCl3) containing tetramethylsilane (TMS) as a chemical shift reference. The ^1^H spectra were obtained at a frequency of 400.18 MHz with a relaxation delay of 4.0 s, an acquisition time of 2.6 s, and 16 scans.

## 3. Results

### 3.1. Multi-Stream Fractionation of PET

The concept of plastic waste fractionation for multi-stream usage is as shown in Figure 1. The research focuses on PET, which accounts for 12% of total solid waste [45]. We hypothesized that hydrolysis-based treatment method can effectively downsize the PET particles, with a generation of different PET fractions for multi-stream usage based on their forms, achieving PET upcycling in a way that is both efficient and environment-friendly. To investigate the hypothesis, we treated PET materials with water in an alkaline environment at 121 °C for 1 h using a laboratory autoclave instrument and separated the soluble and insoluble fractions after the treatment. The insoluble fraction of hydrolyzed PET was utilized as an effective modifier to improve asphalt binder performance, and the soluble fraction was utilized as a carbon source for microbial fermentation to produce lipids for biodiesel (Figure 1).

### 3.2. Solubilized PET for Microbial Lipids Production

**Figure 2:**
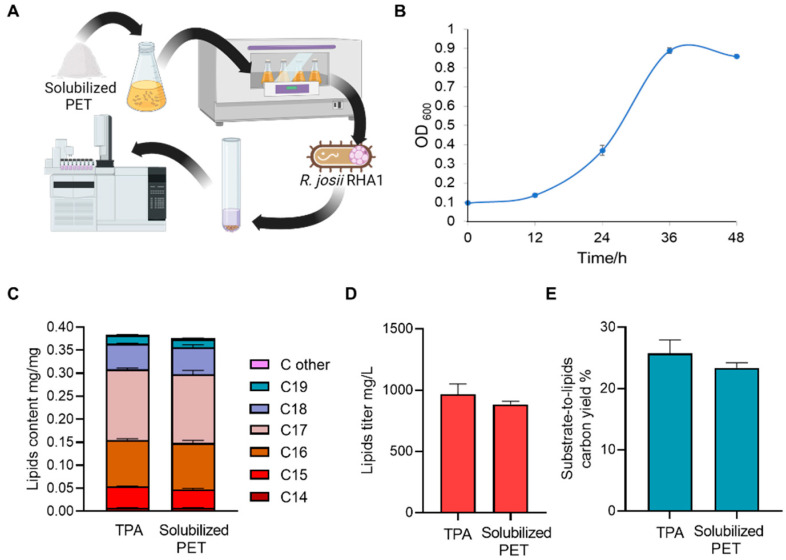
Solubilized PET after alkaline-assisted fractionation support cell growth of *R. jostii* RHA1 and lipid fermentation. (**A**) Illustrations depicting the microbial utilization of solubilized PET and lipid analysis. (**B**) Growth curve of RHA1 indicated by OD_600_ in minimum medium with the solubilized PET or TPA as the sole carbon source. (**C**–**E**) Lipid components and contents (**C**), lipid titer (**D**), and carbon yield of lipids (**E**) obtained with *R. jostii* RHA1 from solubilized PET or TPA. All data were collected with three biological triplicates, processed with GraphPad Prism 9, and shown as mean ± SEM (standard error of mean).

Biodiesel, which can be produced from microbial lipids, has been proposed as a sustainable alternative to replace or supply fossil fuels in certain applications [46,47]. The predicted terephthalate bioconversion pathways of bacteria *R. jostii RHA1*, which is a oleaginous microorganism, inspired us to explore the possibility of upcycling PET soluble fractions to lipids for biodiesel production [48,49]. First, we extracted and analyzed the soluble fraction using GC/MS [36] and found that the PET monomer, TPA, is the dominant depolymerization product (Figure 2A and Figure S1). This suggested that the alkaline hydrolysis specifically generated high-purity monomers, which is essential for the subsequent bioconversion.

Second, we then tested if the solubilized PET could support the growth of oleaginous bacteria *R. jostii* RHA1. With the solubilized PET as the sole carbon source, the cell density of *R. jostii* RHA1 indicated by the optical density at a 600 nm wavelength (OD_600_) increased from 0.097 ± 0.002 to 0.860 ± 0.006 in 36 h (Figure 2B). This growth rate is comparable to that with typical carbon sources like glucose [50]. The result suggested that the fractionated soluble PET can be used as a substrate to efficiently support RHA1 growth. Lastly, we investigated the lipid production of RHA1 with the solubilized PET as a carbon source. The results showed that the lipid content (lipids/DCW *w/w*) in RHA reached 37.0 ± 1.5%, which is about the same level as the RHA cells that utilized pure TPA (37.6 ± 0.7%), supporting that the major component of the solubilized PET is TPA (Figure 2C). A component comparison of the lipids from the two carbon sources also showed similar results, revealing that 15–18 carbon fatty acids constituted the predominant components, comprising 83.4 ± 8.1% and 82.5 ± 4.3%, respectively (Figure 2C). These results demonstrated that *R. jostii* RHA1 utilized the fractionated PET and TPA as the sole carbon source to support their growth and lipid accumulation. To further evaluate the viability of fractionated PET as a feedstock for lipid production, we calculated the lipid titer, which was found to reach 884.2 ± 20.7 mg/L, also comparable to 966.9 ± 67.9 mg/L from TPA and other typical industrial lipid-producing microorganisms [51,52] (Figure 2D). Furthermore, the carbon yield from the solubilized PET and TPA to lipid were 23.4 ± 0.7% and 25.7 ± 0.2%, respectively (Figure 2E). Overall, the results validated our hypothesis that the soluble fraction can be efficiently utilized as a carbon source for biodiesel precursor lipids production. Additionally, our findings supported a previous transcriptomics analysis revealing the existence of the terephthalate degradation pathway in the *R. jostii* RHA1, and for the first time demonstrated that *R. jostii* RHA1 can utilize fractionated PET as the sole carbon source for growth and lipid production [48].

### 3.3. Characterization of Insolubilized PET and Modified Asphalt Binder

**Figure 3:**
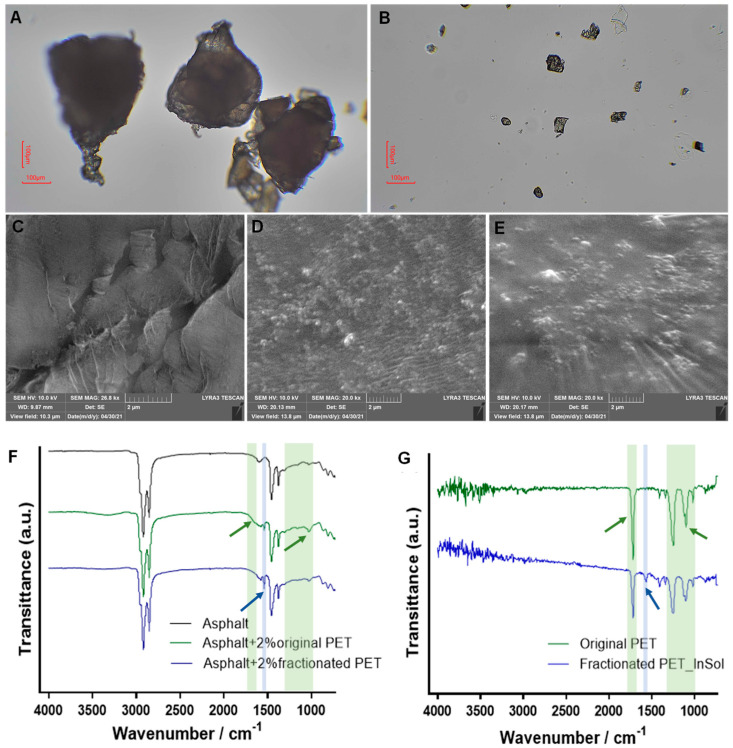
Characterizations of the original and fractionated PET, as well as the original and PET-modified asphalt binders. (**A**,**B**) Optical microscope images of original (**A**) and fractionated PET particles (**B**). (**C**–**E**) SEM images of original asphalt binder (**C**) and modified asphalt binders (**D**,**E**) with 5% original and fractionated PET, respectively. (**F**,**G**) FTIR spectra of asphalt binders (**F**) and PET particles (**G**), with characteristic peaks highlighted. Green arrows denote original PET; blue arrows denote fractionated PET.

To determine whether the hydrolysis-based fractionation effectively downsized the PET particles, the original and fractionated PET particles were compared under optical microscopy, and the images showed that the size of the PET particles decreased significantly after fractionation, from about 300 µm to about 30 µm (Figure 3A,B). This result, combined with the presence of PET monomers in the soluble fraction, supports the hypothesis that hydrolysis-assisted fractionation is an efficient method for downsizing PET materials while preventing the release of microplastics. Notably, our hydrolysis-based fractionation method, compared to conventional alkaline hydrolysis processes, used a lower pressure, temperature, and NaOH concentration, which make the process a “greener” way to recycle PET waste [53]. Additionally, unlike other PET chemical recycling methods such as methanolysis, glycolysis, and aminolysis, it produces RHA1 strain utilizable TPA and avoided the involvement of additional hazardous substrate substances like methanol or amines that could be toxic to microbial lipid production [54].

Original and fractionated PETs (2% and 5%) were added into the pre-melt asphalt binder and evenly stirred to produce PET-modified asphalt binders (Figure 3C,D). To assess the uniform dispersion of PET particles within the binder and investigate the microstructure of the modified asphalt binder, an SEM was employed to examine the morphology of different asphalt samples. Microscopic examination of the original asphalt indicated a non-smooth surface with certain disconnections (Figure 3E). These characteristics have the potential to contribute to pavement deformation under challenging conditions [55]. In the PET-modified asphalt samples, PET additives are observable as tiny white particles in the images that showed a good dispersion state in the modified asphalt binders (Figure 3F,G). Notably, the two modified asphalt binders exhibited smoother micrographic surfaces than the original asphalt binder, highlighting the effect of PET incorporation on the cohesion of the asphalt mixture [56].

Fourier Transform Infrared (FTIR) analysis was conducted to identify chemical bonds and functional groups in the asphalt binders, both unmodified and modified with PET. Characteristic peaks of the asphalt binder at 2919, 2850, 1459, and 1375 cm^−1^ were identified [57]. The introduction of PET additives was evident through strong stretching vibration bands at 1031 cm^−1^, representing the ester group bond C–O observed in both PET particles and modified asphalt binders [57] (Figure 3F,G). Specifically, the characteristic peak of PET at 1730 cm^−1^ was present in the modified binders (Figure 3F,G). The intensity of these PET characteristic peaks was lower in the fractionated-PET-modified asphalt binder compared to the original-PET-modified binder, aligning with the observed pattern in fractionated and original PET samples (Figure 3F,G). This consistency suggests that the observed peaks in PET-modified binders primarily originated from PET particles rather than interactions between PET and asphalt binder. In contrast, the peak observed at 1540 cm^−1^, indicative of reactive aromatic groups, exhibited an opposing trend, displaying higher intensity in the fractionated-PET-modified binder compared to the original-PET-modified binder [58] (Figure 3F,G). Notably, this aromatic group peak was detected in the fractionated PET but not in the original PET (Figure 3G, blue area). These findings suggested that the aromatic group peak in the modified binders is influenced by both PET incorporation and the interaction between PET microparticles and the asphalt binder, involving the aromatic structures inherent in PET polymers [59].

### 3.4. Insolubilized PET for Asphalt Binder Modification

**Figure 4:**
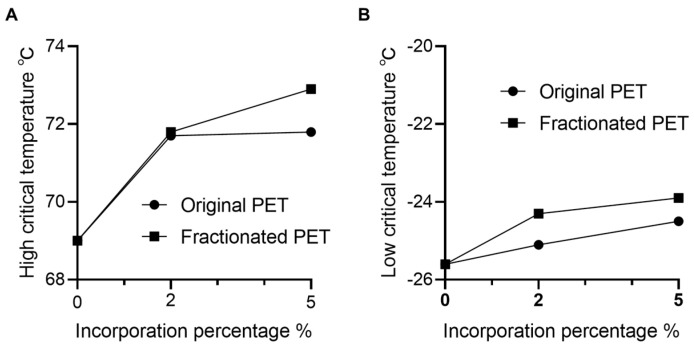
Effect of PET particle incorporation on asphalt binders’ performance. (**A**) High-temperature performance of the asphalt binder modified with original and insoluble PET. The high critical temperature is determined by the rutting factors (G*/sin δ) of the asphalt binder specimens, reaching an equilibrium value of 1.0 kPa in the DSR test. (**B**) Low-temperature performance of the asphalt binder modified with original and insoluble PET. The determination of the low critical temperature in the BBR test relies on the relaxation constant (m) and flexural creep stiffness (S) at 60 s of loading, set at 0.300 and 300 kPa, respectively. Data was processed with GraphPad Prism 9.

To evaluate the potential of the PET particles as a modifier to enhance high-temperature rutting resistance of asphalt binders, original or modified asphalt binders were subjected to the DSR test to measure the high critical temperature [40] (Figure 4A). The results showed that the addition of PET and fractionated PET at 2% elevated the high critical temperature from 69 °C to 71.7 and 71.8 °C, respectively, signifying a notable enhancement in the performance grade (PG) of the asphalt binder, progressing from PG 64 to PG 70 after the modification with PET particles. The improved high-temperature performance could be attributed to the rising softening point of asphalt binders with PET polymer addition [60].

It is noteworthy that further increasing the original PET content to 5% did not yield a significant additional improvement in performance temperature. However, escalating the incorporation of fractionated PET to 5% resulted in a further increase in the fall temperature, reaching 72.9 °C (Figure 4A). This difference could be attributed to the larger specific surface area of the smaller-size fractionated PET, leading to an improved cohesion to the binder. Given the potential existence of an optimal incorporation percentage of PET particles for resilient asphalt binder performance, the results indicate that downsized PET particles after fractionation exhibit a higher optimal binder content [20].

Due to the high fusion temperature and relatively rigid nature of PET particles compared to the elastomeric characteristics of asphalt binders, incorporating PET usually increases the stiffness of the binder, making it more prone to cracking in cold temperatures [61,62,63]. To study the effect of fractionated PET in low temperature performance, we conducted the BBR test to determine the low critical temperature of asphalt binders (Figure 4B). Indeed, the increase of both original and fractionated PET up to 5% resulted in an increase in the low critical temperature, from −25.6 °C to −24.5 and −23.9 °C, respectively (Figure 4B). Despite this marginal decline, the asphalt binder’s low-temperature PG level remained at −22, underscoring the efficacy of PET incorporation without substantial compromise to its low-temperature performance. Surprisingly, compared to original PET-modified binder, the fractionated PET-modified binder showed a greater decrease in low critical temperature (Figure 4B). This observation could be ascribed to the fact that fractionated PET particles with smaller sizes tended to form aggregates in certain areas within the asphalt mixture (Figure 3G). The existence of the large sized aggregates can affect the adhesion between the asphalt binder and PET [64,65]. However, smaller particles that have a larger specific surface area can facilitate a uniform mix in asphalt binder, thus decreasing the chance of phase separation and enhancing the stability of the modified asphalt [66,67]. Notably, a recent study demonstrated that the incorporation of micronized PET particles improved the viscosity, adhesion, and resilience of the asphalt mixers [56].

## 4. Discussion

PET plastic waste is typically chemically inert, but the alkaline-assisted fractionation renders two fractions that are amenable to bioconversion and pavement additive applications, respectively. While the current hydrolysis process has successfully generated TPA monomers and reduced the size of PET particles for subsequent upcycling, further investigation on parameters like processing time and alkaline concentration could potentially enhance monomer yield and overall conversion efficiency.

For the subsequent bioconversion, our results have demonstrated an approach to upcycling PET waste to biodiesel precursor lipids. Even though the terephthalate degradation pathways were predicted in *R. jostii* RHA1 through comparative genomics, no study has experimentally demonstrated the bioconversion of plastic waste using this strain. This demonstration highlighted the viability of biodiesel production from PET waste, offering the potential to decrease PET treatment costs and feedstock expenses for lipid bioproduction. The utilization of readily available, low-cost substrates will play a critical role in making biodiesel economically competitive [68]. Furthermore, considering the versatile metabolism of *Rhodococcus* spp. strains and their potential applications in biotechnological strategies for compound production, the study opened new avenues to engineer *R. jostii* RHA1 to convert plastic waste to a broad range of industrial products, including terpenes, proteins, bioplastics, and others [69,70].

For asphalt binder modification, the insoluble fraction of PET improved the high-temperature performance of asphalt binder by 1 PG, from PG 64 to PG 70, without decreasing its low-temperature PG. This improvement could be attributed to the reduced particle size after fractionation. The smaller-sized particles become more amenable to blend in asphalt binders, promoting dissolution and exhibiting aromatic groups [35] (Figure 3F). It could facilitate the interactions between PET polymers and the aromatic complex within the asphalt binders [59]. The incorporation of aromatic groups into the modified asphalt binders was further evidenced by the increase in the relative content of aromatic hydrogens and benzylic protons determined with ^1^H NMR analysis (Table S1). In our prior investigation on a lignin-based asphalt binder modifier, we demonstrated the feasibility of fractionating the polymer and customizing its structure to enhance asphalt performance [34]. Drawing parallels with the molecular structure of lignin, the introduction of PET, which is also characterized by aromatic structures, fostered aromatic interactions with the aromatic structures within asphalt binders, consequently enhancing cohesion and augmenting the stiffness of the binders [71]. Our findings offer a valuable opportunity for delving deeper into the molecular mechanisms that underlie the enhancement of asphalt binder high-temperature performance through the incorporation of plastic waste.

The study also offered an economically feasible solution to address infrastructure resilience in response to the frequent occurrence of extremely high temperatures due to global climate changes. The results are of significant commercial value, as increasing 1 PG of asphalt binder will increase its price by about $100 per ton [72]. A 2% to 5% plastic waste addition will translate into approximately $2000 to $5000 per ton market value of waste plastics. The research provided a practical approach to achieving this economic value, while enhancing the pavement rutting resistance toward rising and fluctuating temperatures. Furthermore, this observation provides an opportunity to investigate the underlying molecular mechanisms in terms of PET-asphalt interaction for improved high-temperature performance.

## 5. Conclusions

The study demonstrated the potential of multi-stream valorization to achieve plastic waste upcycling for both the usage of pavement material additives and biodiesel precursor production. We have shown that *R. jostii* RHA1 converts the soluble fraction of PET efficiently into lipids. We characterized the fractionized PET and the PET-modified asphalt binders with SEM and FTIR to reveal the incorporation of fractionized PET in asphalt and corresponding morphological changes. We have also shown that an addition of the fractioned PET significantly improved the high-temperature performance of asphalt binder by 1 PG without compromising the low-temperature performance. These results showcased the potential of the multi-streamed usage strategy of PET wastes on responding the trendy global warming and sustainable bioenergy generation in the future. Additionally, the enhanced high-temperature performance observed due to the inclusion of PET warranted a more in-depth exploration of the molecular-level interaction mechanisms between PET and asphalt mixers. This exploration, along with future PET treatment method design, will contribute to the utilization of PET for pavement material with enhanced high-temperature performance.

## Data Availability

Data are contained within the article and Supplementary Materials.

## Supplementary Materials

The following supporting information can be downloaded at https://www.mdpi.com/article/10.3390/polym16060796/s1, Figure S1: GC/MS characterization of the solubilized PET; Table S1: Relative contents of aromatic hydrogens and benzylic protons in asphalt binders determined by ^1^H NMR.
