# Supplementary material for: A Multi-Streamline Approach for Upcycling PET into a Biodiesel and Asphalt Modifier
Source: Polymers (Basel). 2024 Mar 13;16(6):796. doi: 10.3390/polym16060796 (PMC10975289; doi:10.3390/polym16060796)
Supplement: Supplementary file 1 [file polymers-16-00796-s001.zip › polymers-2850444-supplementary.pdf]

Supplementary Material for:

A Multi-Streamline Approach for Upcycling PET into Biodiesel and Asphalt Modifier

Kainan Chen[1], Zeinab Mraiza[2], Yunqiao Pu[3], and Jinghao Li[1], Zhihua Liu[1], Arthur J. Ragauskas[3,4,5], Fujie Zhou[2]*, Joshua S. Yuan[1,6]*

[1] Synthetic and Systems Biology Innovation Hub, Department of Plant Pathology and Microbiology, Texas A&M University, College Station, TX 77843, USA
[2] Texas A&M Transportation Institute, Texas A&M University, College Station, TX 77843, USA
[3] Joint Institute for Biological Sciences, Biosciences Division, Oak Ridge National Laboratory, Oak Ridge, TN 37831, USA
[4] Department of Chemical and Biomolecular Engineering, The University of Tennessee, Knoxville, TN 37996-2200, USA
[5] Department of Forestry, Wildlife and Fisheries, Center for Renewable Carbon, Institute of Agriculture, The University of Tennessee, Knoxville, TN 37996-2200, USA
[6] Department of Energy, Environmental, and Chemical Engineering, McKelvey School of Engineering, Washington University in St. Louis, St. Louis, MO 63130-4899, USA

* Correspondence: Fujie Zhou: f-zhou@tamu.edu and Joshua S. Yuan: joshua.yuan@wustl.edu

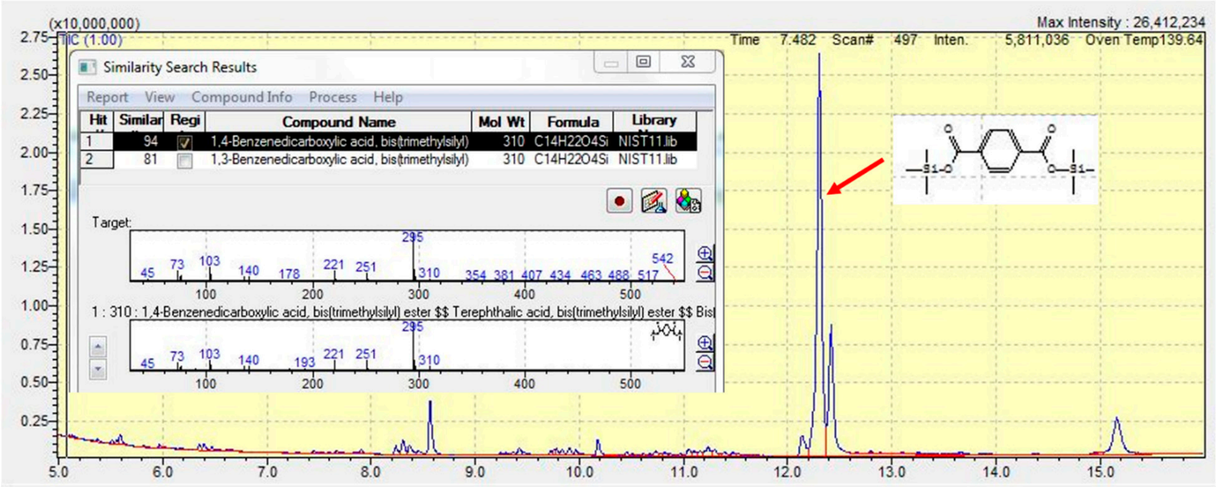

Figure S1. Gas chromatography/mass spectrometry (GC/MS) characterization of the solubilized PET.

Table S1. Relative contents of aromatic hydrogens and benzylic protons in asphalt binders quantified by ^{1}H NMR.

Asphalt samples	Original asphalt	Asphalt modified with 5% original PET	Asphalt modified with 5% fractionated PET
Aromatic H	6.83%	6.95%	7.0%
Benzylic H	12.9%	14.0%	14.1%
